# Clinical factors and comorbidities affecting health-related quality of life in postrenal transplant patients

**DOI:** 10.1097/j.pbj.0000000000000131

**Published:** 2021-06-14

**Authors:** Humera Adeeb, Ihsan Ullah, Mubarak Zeb, Mazharul Haq

**Affiliations:** a Institute of Kidney Diseases MTI HMC Peshawar; b Khyber Medical University Peshawar; c District Health System Peshawar; d Institute of Kidney Diseases MTI HMC Peshawar, Peshawar, Pakistan.

**Keywords:** health-related quality of life, kidney disease component summary, Kidney Diseases Quality of Life-Short Form-1.3, mental component summary, physical component summary, renal transplant

## Abstract

**Background:**

Health-related quality of life is different among different transplant cohorts with respect to different variables which predict mortality and graft survival. The aim of this study was to identify the effects of clinical factors on the health-related quality of life in postrenal transplant patients.

**Methods:**

This census study was conducted at the Institute of Kidney Diseases Peshawar, Pakistan. Data were collected on a questionnaire “ Kidney Diseases Quality of Life-Short Form-1.3 Urdu version” and were analyzed in the 3 main domains, for example, physical component summary (PCS), mental component summary, and kidney disease component summary using SPSS version 21. Mean scores for patients with diabetes mellitus (DM), hypertension, levels of hemoglobin, and serum creatinine were compared by unpaired *t*-test.

**Results:**

A total of 277 men (87.9%) and 38 (12.1%) women participated in the study. Mean age was 37.26 (±10.14) years (range 18–65 years). Hypertension was reported in 72.2% and DM in 10.8%. Hemoglobin was <12.5g% in 26.0% patients. Patients with DM had significant lower PCS (*P* = .001) and mental component summary (MCS; *P* = .001) scores. Patients with hypertension had significant lower MCS score (*P* = .01). Patients with hemoglobin <12.5g% had significantly lower PCS (*P* = .001) score than those with hemoglobin >12.5 g%. The PCS score in patients with serum creatinine level >2 mg% was significantly lower (*P* = .02) than those with serum creatinine <2 mg%.

**Conclusion:**

Lower graft function and DM were associated with lower PCS and MCS scores. Hypertension was associated with lower MCS score and anemia with lower PCS score.

## Introduction

Health-related quality of life (HRQOL) is an important assessment aspect of renal transplant.^1–3^ Chronic kidney disease (CKD) is creeping slowly in the trail of risk factors like hypertension (HTN), diabetes mellitus (DM), renal stone diseases, and glomerulonephritis.^4^ The ultimate end of CKD is end-stage renal disease (ESRD), which is managed with life-long dialysis or renal transplant. Dialysis is time consuming, associated with complications, and access to dialysis is limited in Pakistan. The long-term viable therapeutic management of ESRD is renal transplant.^5^ Compared to dialysis, renal transplant improves survival and quality of life in terms of patient's hope for physical, mental, and social wellbeing despite problems in getting a transplant especially in developing countries.^6–8^

The puzzle of wellbeing is not solved without social and psychological domains. HRQOL measurement translates objective assessment into subjective perspective of the patients who reflects their HRQOL. HRQOL is a dynamic concept that incorporates past experiences, present circumstances, and future expectations into wholesome assessment of health. This concept is operationalized in terms of physical, mental, and social functioning domains.^9^ Although the HRQOL advantages of renal transplantation are well established, large differences of quality of life are often observed depending on specific transplant cohorts.^10^ HRQOL independently predicts mortality, survival, and renal graft failure.^1–3,11^

The prevalence of ESRD in Pakistan is reported as 100 patients per million populations (ppmp). Out of this population only 10% have access to renal supportive therapy (RST) in the form of hemodialysis or peritoneal dialysis.^5^ Asia has the highest number of people entering ESRD. The prevalence of early stages of CKD1, 2 has been reported 5% to 7% worldwide with major contribution to this prevalence from developing countries. Patients from Pakistan and India have lower mean age at the time of initiation of RST as compared to other nations. This age group represents the productive population leading to loss of working people.^12,13^

Renal graft function is maintained with the help of immunosuppressant. Hypertension and diabetes are the 2 major morbidities contributing to ESRD. The ongoing complications associated with these 2 conditions keeps on mounting the risk of target organ damage and of graft failure. Hypertension has been reported the most common condition followed by DM being managed in renal transplant recipients.^14^ Renal impairment affects several body functions and cause symptoms that may reduce health-related quality of life (HRQOL).^10^ Prevalence of hypertension was reported 46.2% in Pakistan and is leading cause of ESRD globally.^13,15^ Prevalence of type 2DM in Pakistan was reported 16.98%.^16^ DM leads to diabetic nephropathy that ultimately ends in ESRD. Anemia is a common condition if overlooked, contributes to graft failure and mortality.^17^

The present study was designed to look into the clinical factors in postrenal transplant patients and their effect on the HRQOL. One of the most important issues for the future of transplantation is to specify the full range of personal, environmental, and clinical factors that negatively influence the HRQOL outcomes. A better understanding of the role of these factors is essential to develop interventions that maximize the HRQOL in these patients. The World Health Organization prioritizes HRQOL improvement for people living with chronic diseases requiring lifelong management.^18^

## Methods

### Study design

It was a cross-sectional study conducted at Department of Nephrology, Institute of Kidney Diseases (IKD) Hayatabad Medical Complex Peshawar, Pakistan.

### Ethical approval

The Study was approved by Khyber Medical University Advanced Studies Research Board in its 62nd meeting on 26th September 2018. Ethical approval was sought from ethical review committee of IKD. Data were collected from October 1, 2018 to January 31, 2019.

### Inclusion criteria

All postrenal transplant patients registered at IKD till first October 2018 were enlisted for inclusion to conduct a census provided they met the inclusion criteria. Patients were approached during their routine monthly follow-up visit. Patients with age 18 years or more, minimum post-transplant period of 16 weeks or more with no infection during past 4 weeks were interviewed.

### Exclusion criteria

Patients with acute graft rejection, currently admitted/remained admitted in the previous 4 weeks for any reason and chronic allograft nephropathy requiring RST were excluded.

### Data collection

Background information about sex, age, education, duration of transplant, previous RST, comorbidity, donor-related or unrelated, relation with the donor in case of related, employment status, and immunosuppressant medication were recorded. Patients were interviewed after obtaining prior training by the investigator according to the instructions mentioned by RAND (http://www.rand.org) in the office setting. Those patients who were able to fill the questionnaire were allowed to do so by themselves. Serum creatinine and hemoglobin were measured in the Department of Pathology, IKD under the supervision of consultant pathologist using automated analyzers by Siemens (Germany) and Fisher Scientific (USA) respectively. Data were collected on a paper questionnaire Kidney Diseases Quality of Life-Short Form (KDQOL SF)-1.3 Urdu version^19^ and analyzed.

### Data analysis

**Figure 1:**
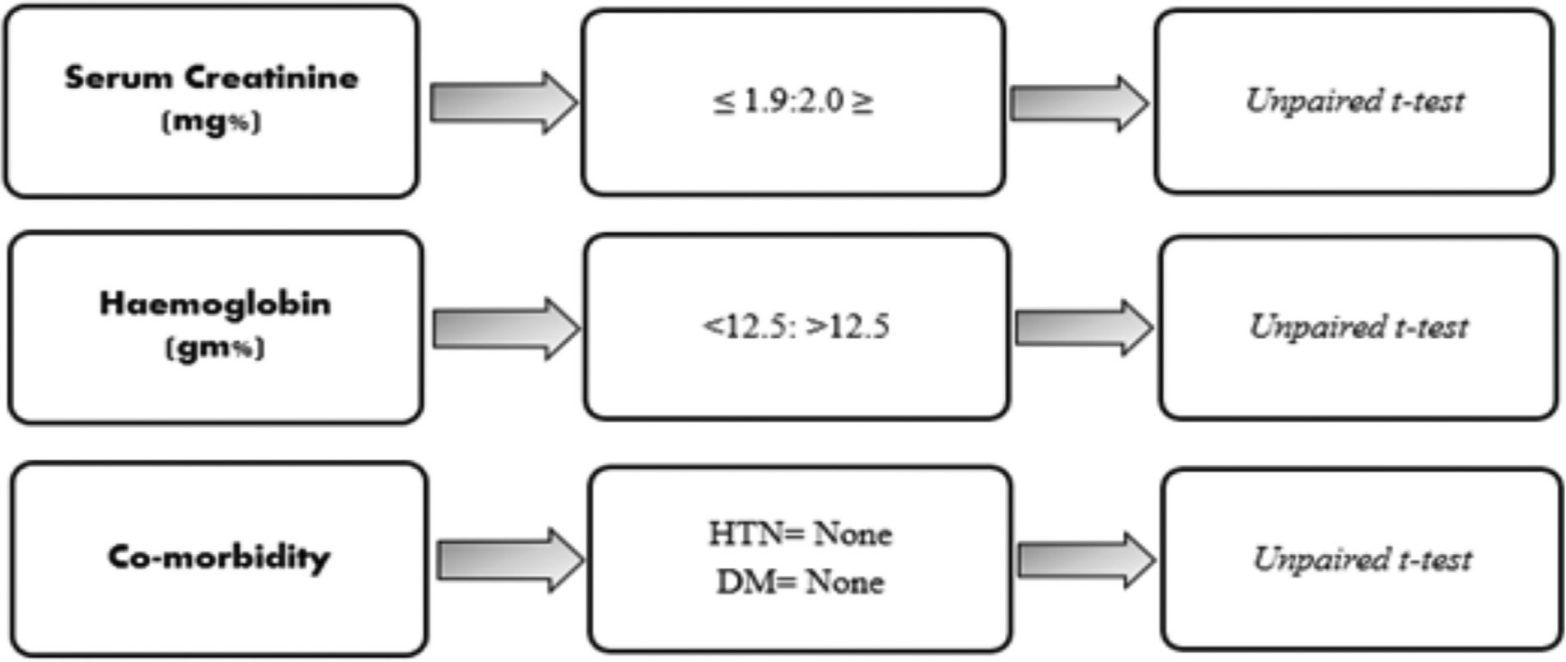
Variables and categories analyzed by unpaired *t* test. DM = diabetes mellitus, HTN = hypertension.

Data were analyzed by SPSS version 21. Mean score for the 3 the domains, kidney disease component summary (KDCS), physical component summary (PCS), and mental component summary (MCS) were calculated. Continuous variables were calculated as mean with standard deviation and categorical variables as frequency and percentages. Unpaired *t* test was applied for comparison of groups with DM, HTN against those without the said conditions, serum creatinine (<2 mg%, ≥2 mg%), and hemoglobin (Hb ≤12.5 g%, >12 g%). These variables were compared across PCS, MCS, and KDCS domains and a *P* value of ≤.05 was considered statistically significant (Fig. 1).

## Results

A total of 277 men (87.9%) and 38 (12.1%) women participated in the study. Age range was18 to 65 years, and mean age was 37.26 (±10.14) years. Majority (177 [56%]) were unemployed. Live related transplant recipients were 130 (42.37%) and the rest were live unrelated transplant recipients. Transplant duration was between 1 and 5 years in 196 (62.22%), 4 months to 1 year in 44 (13.9%), 5 to 10 years in 45 (14.2%), and >10 years in 30 (9.5%). Mean scores of KDQOL SF-1.3 in its 3 domains PCS, MCS, and KDCS were 44.86 (±4.14), 37.87 (±3.40), and 76.21 (±7.04), respectively. Hypertension was reported in 72.2% and 10.8% had DM. Hemoglobin was <12.5 g% in 26.0% patients and serum creatinine was >2.0 mg% in 24% patients.

### KDQOL SF-1.3 score for three domains according to serum creatinine levels

Postrenal transplant patients with serum creatinine levels >2.0 mg% sored significantly lower in the PCS (*P* value = .02) and MCS (*P* value = .01) scales. There was no significant difference in the KDCS scores as shown in Table 1.

**Table 1:** Kidney Diseases Quality of Life-Short Form-1.3 score for three domains according to serum creatinine levels

	Serum creatinine (mg%)	n	Mean	SD±	*t*	*P*
PCS	<2	254	44.83	2.24	−3.043	.02^∗^
	≥2	61	40.88	8.27		
MCS	<2	254	38.14	3.36	2.51	.01^†^
	≥2	61	36.92	3.56		
KDCS	<2	254	76.17	7.22	−0.13	.89
	≥2	61	76.30	6.26		

KDCS = kidney disease component summary, MCS = mental component summary.

∗ Statistically significant.

† Statically highly significant.

### KDQOL SF-1.3 scores for three domains according to hemoglobin levels of patients

An unpaired *t* test was conducted to compare mean score in postrenal transplant patients with hemoglobin <12.5 g% and ≥12.5 g%. In the PCS domain patients with hemoglobin <12.5 g% had significantly lower score (*P* value = .001) than those with hemoglobin >12.5 g%. In the MCS and KDCS category, no significant difference was found as shown in Table 2.

**Table 2:** Group statistics and *t* test for three domains according hemoglobin categories

	Hemoglobin (g %)	n	Mean	SD±	*T*	*P*
PCS	<12.5	82	43.38	4.78	−3.785	.001^†^
	≥12.5	233	45.35	3.76		
MCS	<12.5	82	37.61	3.21	−0.907	.36
	≥12.5	233	38.01	3.50		
KDCS	<1	82	76.87	7.98	1.010	.31
	≥12.5	233	75.95	6.68		

† Statically highly significant. KDCS = kidney disease component summary; MCS = mental component summary.

### KDQOL SF-1.3 scores comparison for three domains according to comorbidity “diabetes mellitus and hypertension”

An unpaired *t* test was conducted to compare the mean scores of postrenal transplant patients with DM and those who were neither diabetics nor hypertensive. There was a significant difference in PCS domain between DM and no comorbidity (*P* value = .001), but no such difference was seen with HTN in PCS domain. In the MCS domain, DM (*P* value = .001) and HTN (*P* value = .01) showed statistically significant difference as compared to those with no comorbidity. In the KDCS domain, there was no significant difference in the score as shown in Table 3.

**Table 3:** Group statistics and *t* test for the three domains according to diabetes mellitus and hypertension

	Comorbidity	n	Mean	SD±	*t*	*P*
PCS	DM	34	44.02	1.94	−3.07	.001^∗^
	None	30	41.07	5.43		
	HTN	228	45.04	4.21	1.13	.25
	None	30	44.07	5.43		
MCS	DM	34	34.42	5.53	−3.01	.001^∗^
	None	30	36.19	3.97		
	HTN	228	33.74	4.80	2.51	.01^†^
	None	30	36.79	3.97		
KDCS	DM	34	74.76	4.41	−0.17	.86
	None	30	75.03	7.87		
	HTN	228	76.31	7.32	0.89	.37
	None	30	75.03	7.87		

∗ Statically highly significant.

† Statistically significant. DM = diabetes mellitus, HTN = hypertension, KDCS = kidney disease component summary, MCS = mental component summary.

## Discussion

The measurement of health has expanded beyond the physical and laboratory parameters by physicians. The WHO included mental and social impacts of a disease along with physical to complete the total horizon of health evaluation.^20^ Incorporation of mental and social domains of health into the definition of health led to evaluation of health in terms of HRQOL. Health is a continuum with physical parameters at one end, but this continuum is completed at the other end by HRQOL issues.^21^ HRQOL is a dynamic concept able to translate the subjective perception into objective measurable physical, mental, and social domains.^9^

The 2 most common comorbidities that are the cause of ESRD and are managed in renal transplant patients are HTN and DM. Patients are on multiple medicines for HTN and DM along with the immunosuppressive therapy after a renal transplant. The ongoing complications associated with these 2 conditions keeps on mounting the risk of target organ damage and of graft failure. Hypertension has been reported the most common condition followed by DM being managed in renal transplant recipients. Hypertension was reported as 87.40% and DM as 21.53%. Presence of comorbidity was associated with lower MCS score.^22^ In a study with 76% hypertensive (*P* = .01) and 18% DM (*P* = .001) patients, patient's PCS scores were significantly different with these comorbidities.^14^

Another study reported no difference in the HRQOL scores of patients with these comorbidities.^23^ HRQOL scores were lower in the postrenal transplant patients having associated comorbidities, and DM was also associated with unemployment as compared to HTN.^24^ These 2 conditions are not only the leading causes of ESRD but also develop as a side effect of the immunosuppressive therapy in postrenal transplant patients. Therefore, possibility of these side effects should be considered when prescribing immunosuppressive therapy in these patients.^14^

Postrenal transplant patients with serum creatinine >2.0 mg% sored significantly lower in PCS and MCS domains. There was no significant difference in KDCS domain between those with serum creatinine <2.0 mg% or >2.0 mg%. The rise in serum creatinine is one of the factor associated with worsening of HRQOL scores. Serum creatinine rise is one of the factor increasing patient as well as physician concern for the graft survival. Apart from its clinical manifestations, it was associated with worse self-reported outcome (KDQOL SF-1.3) after transplant. All HRQOL scores were inversely associated with increasing serum creatinine.^10^

While based on estimated glomerular filtration rate, HRQOL (Medical Outcome Short Form (MOSF)-36) scores were significantly affected by creatinine clearance in the role physical, general health, role emotional, and vitality subscales.^25^ Patients with high serum creatinine reported lower HRQOL (MOSF-36) scores in PCS domain.^1^ Patients with serum creatinine >2.0 mg% scored in PCS domain lower than those with serum creatinine of <2.0 mg%.^26^ Rising serum creatinine was inversely associated with physical functioning, general health, social functioning and vitality, and was statistically significant (*P* = .001).^14^ Patients with serum creatinine of >2.8 mg% had lower HRQOL scores in physical domain as compared to patients with low serum creatinine levels.^27^

In the PCS category, those patients with hemoglobin levels <12.5 g% had significantly lower score (*P* = .001) than those with hemoglobin >12.5 g%. In the MCS and KDCS category, there was no significant difference between the 2 groups. Lower hemoglobin levels were found to be associated with significantly (*P* = .01) lower HRQOL scores in general health, physical functioning, and social functioning.^14^ Similarly, lower HRQOL (MOSF 36) score was found at lower hemoglobin in PCS domain.^1^ Anemia was reported to be a significant predictor of graft failure and mortality during 4-year follow-up of the renal transplant recipients.^17^

The quality of life has been reported to be an equally important parameter in the evaluation of treatment outcome. Imperative in the need to identify are the factors that bring changes in the HRQOL scores. Identification of these factors will help to focus pertinently on their monitoring and management to improve the overall HRQOL scores. Identification of these factors will not only help postrenal transplant patients to optimize their management but also help prospective renal transplant patients to envision their expectations of a kidney transplant.

## Conclusion

In conclusion of the present study, patients having lower graft function and DM were associated with lower PCS and MCS scores, whereas those with HTN was associated with lower MCS score. Those patients presented with anemia were having lower PCS scores.

## Limitations

- The study duration was short, so a cross-sectional study was conducted, which could not predict the trajectory of change in HRQOL.
- Only 1 HRQOL tool was used due to the availability of only 1 validated tool in the national language.
- Generalizability of the findings is limited due to the small number of female patients in the study population.

## Acknowledgments

The author acknowledges all participants and patients who made this study possible.

## Conflicts of interest

The authors declare no conflict of interest with anyone.
